# CAR-T cell therapy in non-Hodgkin lymphoma: a clinical trial landscape review

**DOI:** 10.3389/fimmu.2026.1820425

**Published:** 2026-05-08

**Authors:** Jinyu Wu, Yun Liao, Wen Wang, Xin Zhang

**Affiliations:** 1 Department of Gastroenterology, The First People’s Hospital of Shuangliu District (West China Airport Hospital of Sichuan University), Chengdu, Sichuan, China; 2 Stem Cell Immunity and Regeneration Key Laboratory of Luzhou, Sichuan, Luzhou, China; 3 Department of Laboratory Medicine, The Second Affiliated Hospital of Chengdu Medical College, Nuclear Industry 416 Hospital, Chengdu, Sichuan, China

**Keywords:** chimeric antigen receptor T cell, clinical trial landscape, immunotherapy, molecular targets, non-Hodgkin lymphoma

## Abstract

**Background:**

Chimeric antigen receptor T cell (CAR-T) therapy has emerged as a transformative treatment modality for selected subtypes of non-Hodgkin lymphoma (NHL). While multiple CAR-T products have demonstrated remarkable clinical activity, the overall clinical development landscape remains heterogeneous, with substantial variation in trial design, target selection, geographic distribution, and endpoint prioritization. A comprehensive analysis of the global clinical trial landscape is essential to contextualize current progress and identify unmet needs in this rapidly evolving field.

**Methods:**

We conducted a systematic landscape analysis of CAR-T–related clinical trials for NHL using the Trialtrove database. Interventional trials registered up to December 18, 2025, were retrieved using predefined search criteria. Eligible studies were screened according to standardized inclusion and exclusion criteria. Key trial characteristics, including trial status, phase, geographic location, sponsor type, molecular targets, and reported clinical endpoints, were extracted and analyzed descriptively.

**Results:**

A total of 360 eligible clinical trials were included in the final analysis. The global CAR-T clinical trial landscape in NHL is characterized by rapid expansion and a predominant focus on early-phase development. Trial activity is highly concentrated in a limited number of countries, with academic institutions serving as the primary drivers of clinical investigation. CD19-directed CAR-T therapies dominate the current landscape, although emerging diversification toward alternative targets is evident. Reported study endpoints largely emphasize safety and short-term efficacy, whereas durable clinical outcomes remain less frequently assessed, reflecting the exploratory nature of most trials.

**Conclusion:**

CAR-T therapy development in NHL continues to advance rapidly, driven by academic innovation and expanding preclinical insights. However, persistent challenges related to antigen escape, treatment durability, and trial design remain. Future progress will require the integration of next-generation CAR engineering strategies, broader incorporation of long-term clinical endpoints, and alignment with evolving regulatory and policy frameworks to support sustainable clinical translation.

## 1 Introduction

Non-Hodgkin lymphoma (NHL) constitutes a broad and biologically heterogeneous group of lymphoid malignancies arising from B cells, T cells, or natural killer cells, and remains a major contributor to global cancer-related morbidity and mortality (1). The clinical behavior of NHL varies widely, ranging from indolent subtypes with prolonged survival to aggressive forms associated with rapid progression and poor outcomes. Over the past decades, the incorporation of immunochemotherapy, monoclonal antibodies, and targeted agents has substantially improved survival for selected patient populations (2, 3). However, a significant proportion of patients ultimately experience relapse or develop refractory disease, for whom therapeutic options are limited and long-term disease control remains difficult to achieve (4, 5). These challenges underscore the persistent unmet clinical need for innovative therapies capable of delivering durable and meaningful clinical benefit.

Immunotherapy has reshaped the therapeutic landscape of NHL by leveraging the host immune system to recognize and eradicate malignant lymphoid cells (6). Among the emerging immunotherapeutic modalities, chimeric antigen receptor (CAR) T cell (CAR-T) therapy represents one of the most significant advances in the treatment of B-cell malignancies (7). CAR-T therapy involves the genetic engineering of autologous T cells to express synthetic receptors that enable antigen-specific tumor recognition in a major histocompatibility complex–independent manner. This approach allows CAR-T cells to overcome key mechanisms of immune evasion and to exert potent cytotoxic activity against tumor cells (8). Clinical application of CAR-T therapies targeting B-cell lineage antigens has demonstrated remarkable efficacy in heavily pretreated patients with B-cell NHL, establishing CAR-T therapy as a critical therapeutic option for relapsed or refractory disease (9, 10).

Despite these achievements, the clinical development of CAR-T therapy in NHL remains complex and evolving. Treatment-related toxicities, including cytokine release syndrome and immune effector cell–associated neurotoxicity, necessitate careful evaluation of safety and dosing strategies (11, 12). Moreover, not all patients derive durable benefit from CAR-T therapy, with relapse occurring in a subset of responders. Biological factors such as antigen escape, tumor heterogeneity, limited CAR-T cell persistence, and immunosuppressive tumor microenvironments have been implicated in treatment failure, highlighting the need for continued optimization of CAR design and therapeutic strategies (13). These challenges have prompted extensive clinical and translational research efforts aimed at improving efficacy, durability, and safety.

In parallel, the rapid expansion of CAR-T research has resulted in a growing number of clinical trials exploring diverse antigen targets, CAR constructs, and therapeutic strategies across NHL subtypes (14). The pace of development and the diversity of ongoing investigations reflect both the promise of CAR-T therapy and the uncertainties surrounding optimal clinical implementation (15). As clinical trial activity continues to increase, a comprehensive understanding of the global clinical development landscape becomes increasingly important. Clinical trial landscape analyses provide a systematic framework to integrate fragmented trial-level information, enabling assessment of developmental maturity, identification of research priorities, and recognition of gaps in clinical investigation.

Accordingly, a structured evaluation of registered clinical trials investigating CAR-T therapy for NHL is essential to contextualize current progress and inform future research directions. In this study, we conducted a comprehensive landscape analysis of CAR-T clinical trials in NHL, systematically characterizing key aspects of clinical development, including trial phase distribution, molecular target selection, geographic and sponsorship patterns, and clinical endpoint strategies. By providing an integrated overview of the current clinical trial landscape, this analysis aims to support rational trial design, guide translational research efforts, and facilitate the continued evolution of CAR-T–based immunotherapy for NHL.

## 2 Methods

### 2.1 Data source and selection criteria

Clinical trial data were retrieved from the Trialtrove database (https://clinicalintelligence.citeline.com/), an authoritative clinical trial intelligence platform that integrates information from multiple global registries, including ClinicalTrials.gov and the World Health Organization International Clinical Trials Registry Platform. We searched for all clinical trials registered up to December 18, 2025 that investigated chimeric antigen receptor T cell (CAR-T) therapy for non-Hodgkin lymphoma (NHL). The search strategy applied the following criteria: Drug type (“CAR-T cell” OR “chimeric antigen receptor T cell”) AND Therapeutic area (“Oncology: Lymphoma, Non-Hodgkin’s”). To ensure data relevance and consistency, only interventional clinical trials were considered eligible for inclusion.

### 2.2 Inclusion and exclusion criteria

Trials were included if they met all of the following criteria: (1) investigated CAR-T cell–based therapies as an intervention; (2) enrolled patients diagnosed with non-Hodgkin lymphoma; (3) were registered as interventional clinical studies; and (4) provided sufficient information on trial characteristics, including trial phase or study objectives. Trials were excluded if they met any of the following criteria: (1) lacked a clearly defined CAR-T target; (2) did not specify the geographic location or country of conduct; (3) were classified under nonstandard or ambiguous trial phase categories; or (4) were observational studies, preclinical studies, or non–CAR-T–based interventions. When multiple records referred to the same clinical trial, duplicate entries were identified and consolidated to avoid redundancy.

### 2.3 Handling incomplete data

Trials with partially missing or incomplete information were retained when core trial attributes were available and could be reliably categorized. Variables with missing values were labeled as “unspecified” and included only in descriptive analyses where appropriate. No imputation was performed for missing data. Sensitivity checks confirmed that the presence of incomplete records did not materially affect the overall trends observed in the landscape analysis.

### 2.4 Subtype classification and heatmap generation

For the subtype–target heatmap, trial records were curated by extracting the primary CAR-T target(s) and the objective/indication text; entries with missing or non-informative target labels were removed. Only records explicitly related to NHL were retained. NHL subtypes were assigned using rule-based keyword matching applied to the objective text, including diffuse large B-cell lymphoma (DLBCL), follicular lymphoma (FL), small lymphocytic lymphoma/chronic lymphocytic leukemia (SLL/CLL), peripheral T-cell lymphoma (PTCL), cutaneous T-cell lymphoma (CTCL), and primary central nervous system lymphoma (PCNSL); when a specific subtype was not stated, entries were categorized into predefined “unspecified” buckets to avoid over-inference. Targets were harmonized to canonical labels by merging synonyms and converting long-form target names to commonly used gene symbols/aliases; multi-target regimens were handled using multi-label assignment, with identical target–subtype pairs counted once per trial record to prevent double counting. Targets were ranked by overall frequency and a reduced set of the most frequent targets was visualized as a contingency heatmap in R (ggplot2), with cell values overlaid and zero cells left blank for readability.

### 2.5 Data extraction and quality control

**Figure 1:**
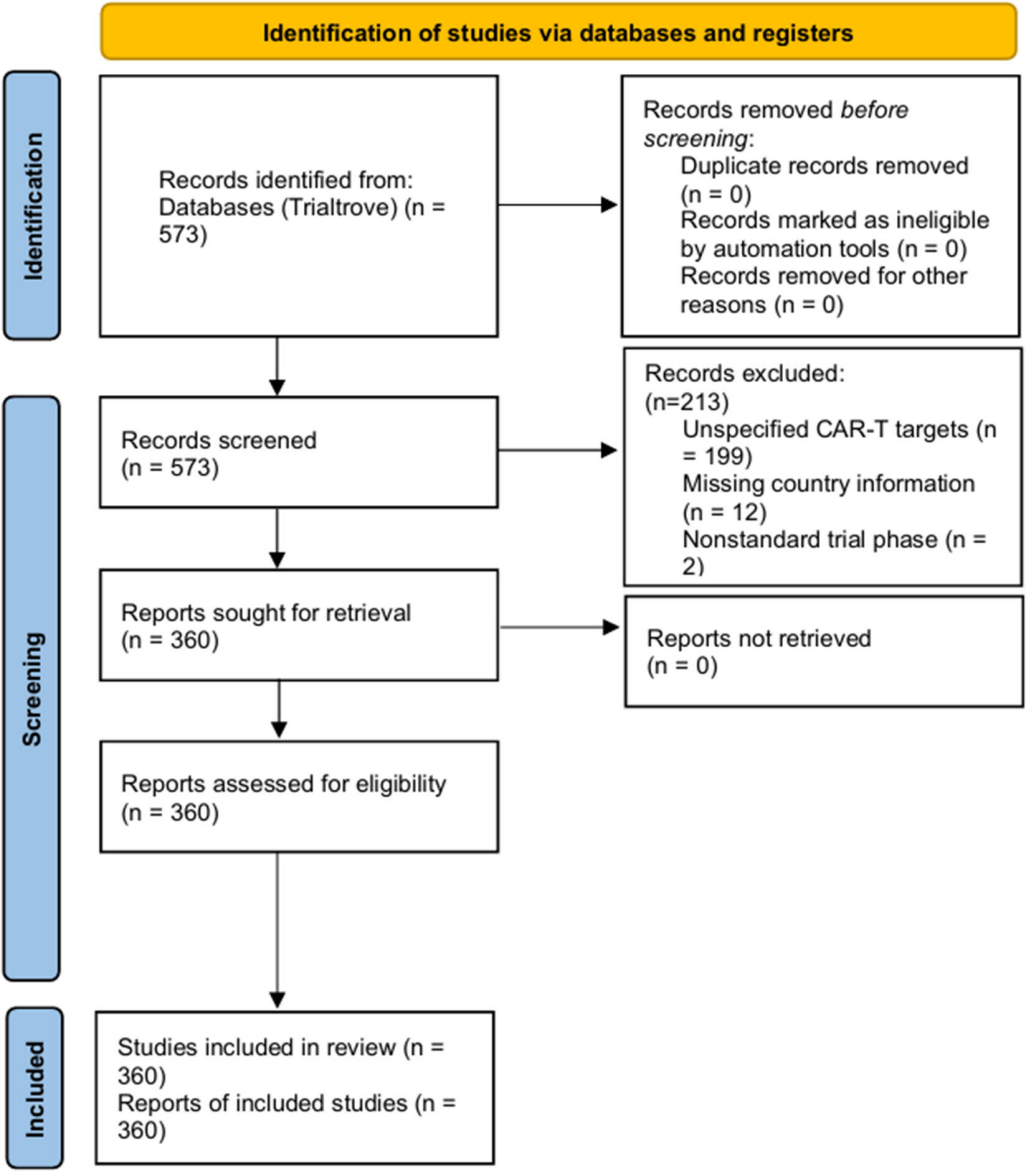
PRISMA 2020 flow diagram of study selection. The diagram illustrates the identification, screening, and inclusion process of clinical trials in this registry-based landscape analysis. A total of 573 records were identified from the Trialtrove database. After screening, 213 records were excluded due to unspecified CAR-T targets (n = 199), missing country information (n = 12), or nonstandard trial phase (n = 2). Ultimately, 360 studies were included in the final analysis. The flow diagram was adapted from the PRISMA 2020 framework to reflect a registry-based study design.

Two independent investigators performed data extraction and cross-validation using a predefined data collection framework. Extracted variables included trial status, phase, geographic location, sponsor type, molecular targets, and reported clinical endpoints. Sponsor-related variables (including sponsor type and role) were extracted directly from structured fields in the Trialtrove database. Sponsor categories used in this study reflect the original database classification and were not independently defined by the authors. Patient-level characteristics (e.g., age, disease stage, and prior treatments) were not systematically extracted due to inconsistent reporting and limited availability in registry-based data sources. Discrepancies were resolved through discussion and consensus to minimize classification bias, particularly when handling unstructured or inconsistently reported data. Following quality control procedures, residual limitations related to data heterogeneity were deemed unlikely to substantially influence the overall conclusions. The study selection process was reported in accordance with the PRISMA 2020 guidelines, adapted for a registry-based clinical trial landscape analysis. A PRISMA flow diagram is presented in **Figure 1**, and the completed PRISMA 2020 checklist is provided in **Supplementary Table S1**.

## 3 Results

### 3.1 Overall landscape and temporal trends

In this study, we systematically reviewed clinical trials evaluating chimeric antigen receptor T-cell (CAR-T) therapies for non-Hodgkin lymphoma (NHL) that were registered in the TrialTrove database up to December, 2025. A total of 573 clinical trials were initially identified. After applying predefined exclusion criteria, we excluded 199 trials with unspecified CAR-T targets, 12 trials lacking country information, and 2 trials categorized as “other” with respect to trial phase. Consequently, 360 clinical trials met the inclusion criteria and were included in the final analysis (**Figure 1**). Detailed characteristics of all included trials are provided in **Supplementary Table S2**.

**Figure 2:**
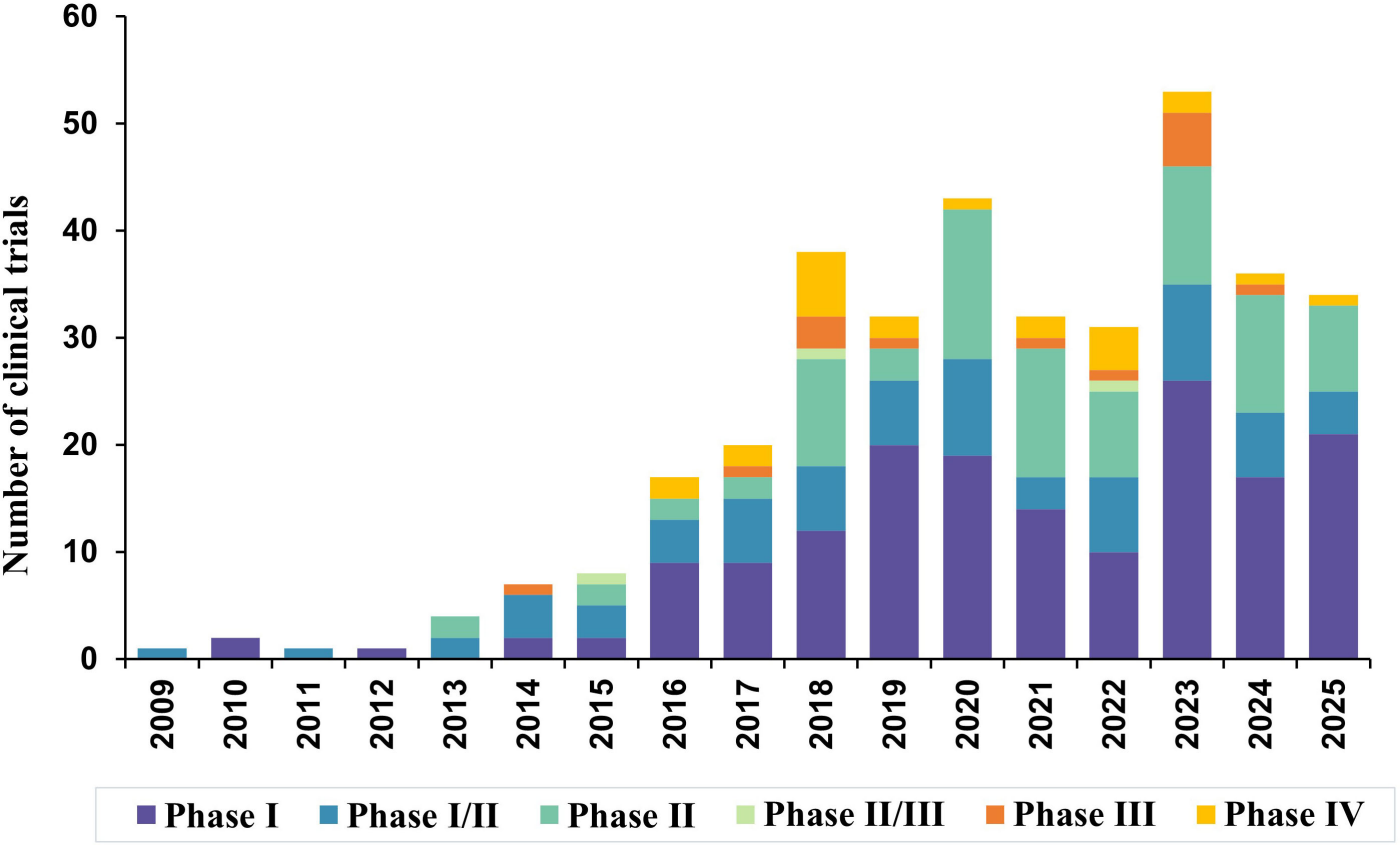
Annual distribution of CAR-T clinical trials in non-Hodgkin lymphoma by trial phase (2009–2025). The stacked bar chart shows the number of clinical trials initiated each year, stratified by trial phase (Phase I, Phase I/II, Phase II, Phase II/III, Phase III, and Phase IV). The data demonstrate a marked increase in CAR-T clinical trials over time, with a predominance of early-phase studies.

The registration of CAR-T–related clinical trials in NHL began in 2009 and remained limited during the early years. From 2014 onward, the number of newly registered studies increased steadily, followed by a marked acceleration after 2016, with trial activity peaking between 2020 and 2023 and remaining sustained through 2025 (**Figure 2**). Across all included studies, early-phase trials predominated, with Phase I studies representing the largest proportion, followed by Phase I/II and Phase II trials. In contrast, late-phase development was relatively limited, with only a small number of Phase II/III, Phase III, and Phase IV trials initiated during the study period (**Figure 2**).

**Figure 3:**
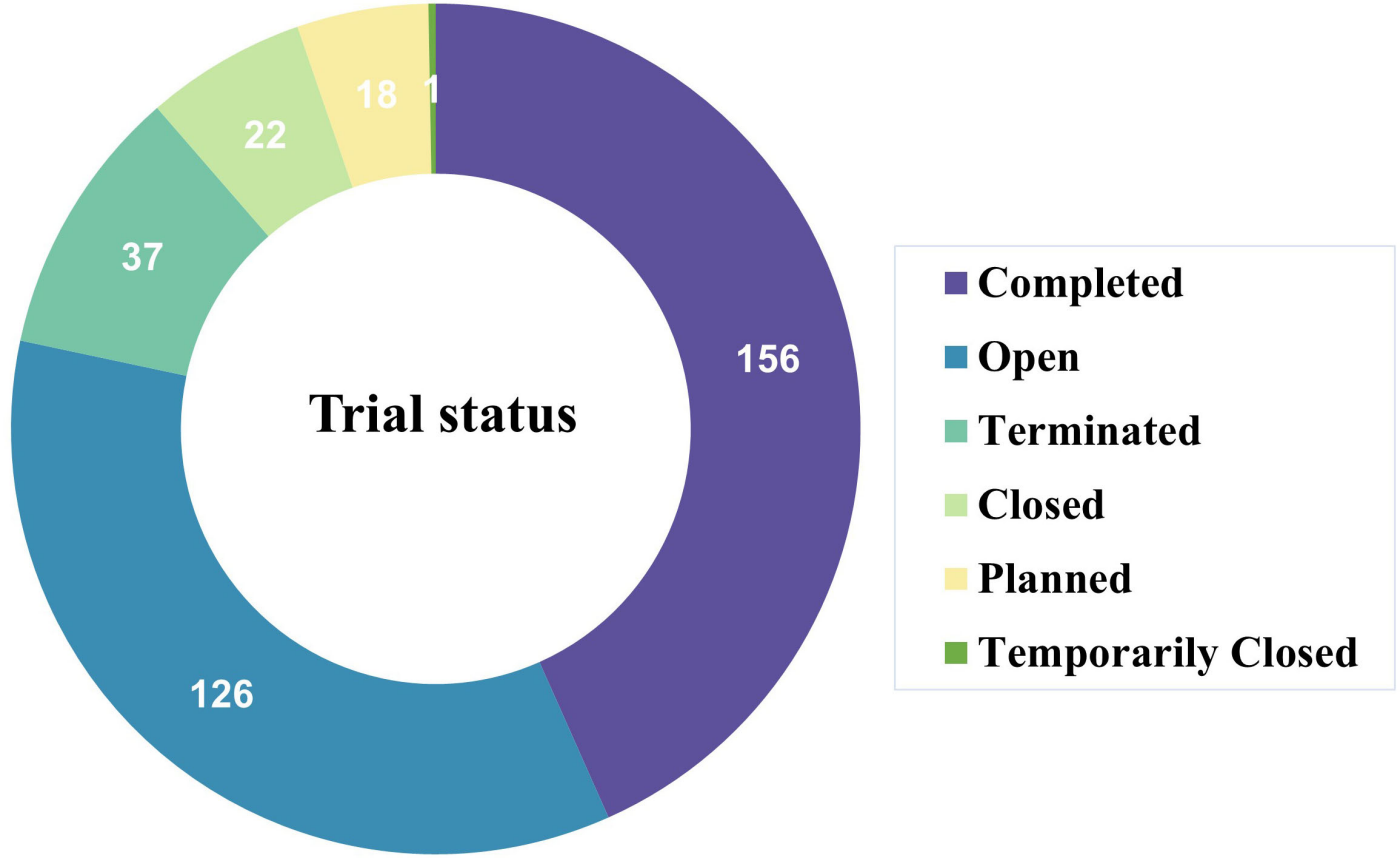
Distribution of trial status among registered CAR-T clinical trials for non-Hodgkin lymphoma. The donut chart summarizes the operational status of the included trials identified from the Trialtrove database. Most studies were classified as completed or actively open, whereas smaller proportions were terminated, closed, planned, or temporarily closed. These findings reflect the ongoing expansion and dynamic evolution of CAR-T clinical development in NHL.

Regarding trial status, 156 studies were completed, while 126 trials were ongoing, indicating both substantial accumulated clinical experience and continued investigational momentum in the CAR-T field for NHL (**Figure 3**). Overall, these findings delineate a rapidly expanding yet predominantly early-phase clinical trial landscape for CAR-T therapies in NHL.

### 3.2 Geographic distribution and sponsor characteristics

**Figure 4:**
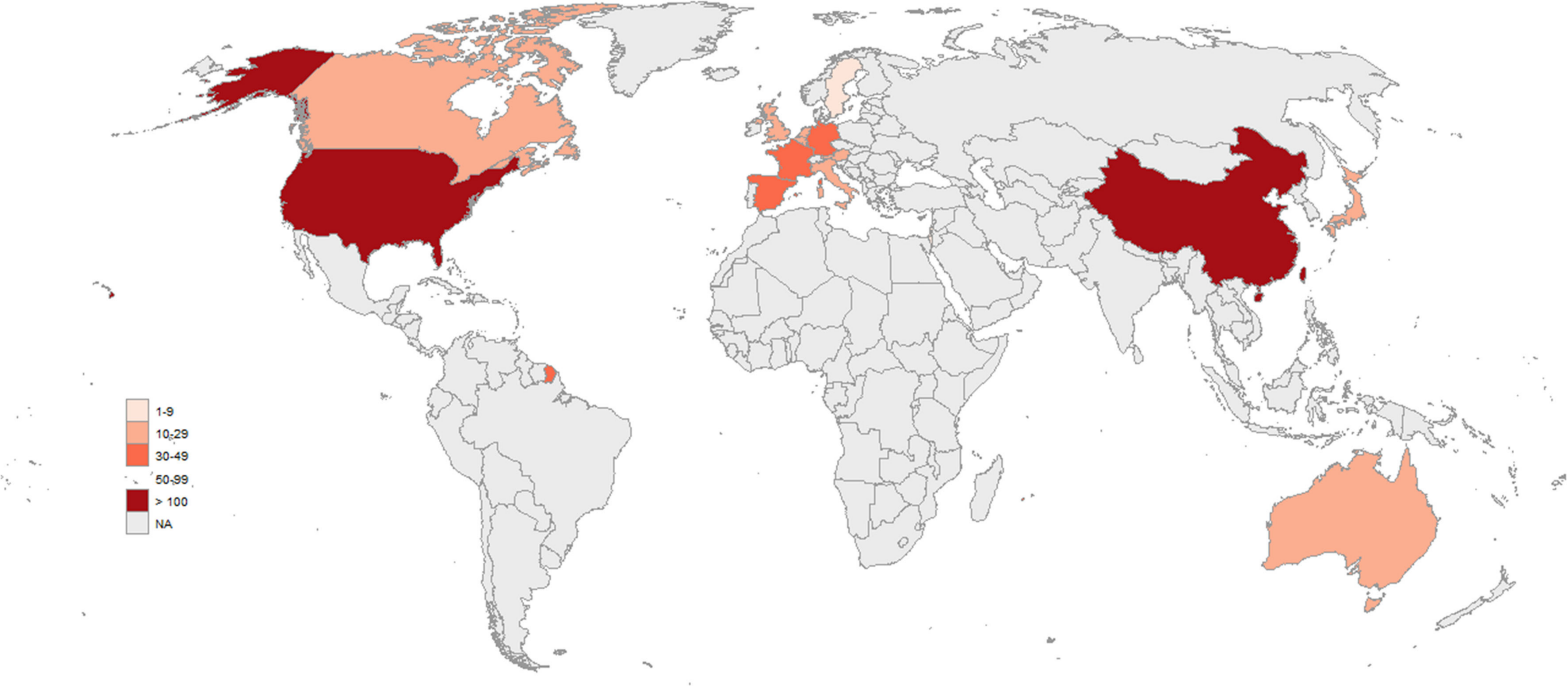
Global geographic distribution of CAR-T clinical trials. The world map displays the number of registered clinical trials across countries. Color intensity represents trial density, categorized into predefined ranges. The majority of trials are concentrated in the United States and China, with additional contributions from Europe and other regions. The map presents the global distribution of all recorded trials, including regions not highlighted in the top-country analysis.

The global distribution of CAR-T clinical trials in non-Hodgkin lymphoma demonstrates a pronounced geographic imbalance, with trial activity concentrated in a limited number of regions. Overall, CAR-T studies are predominantly conducted in East Asia, North America, and parts of Western Europe, while large regions of Africa and the Middle East show minimal or no registered trial activity. This spatial pattern highlights substantial regional disparities in CAR-T clinical research engagement at the global level (**Figure 4**). While the global map includes all recorded trial locations, the main analysis focuses on the leading countries by trial count.

**Figure 5:**
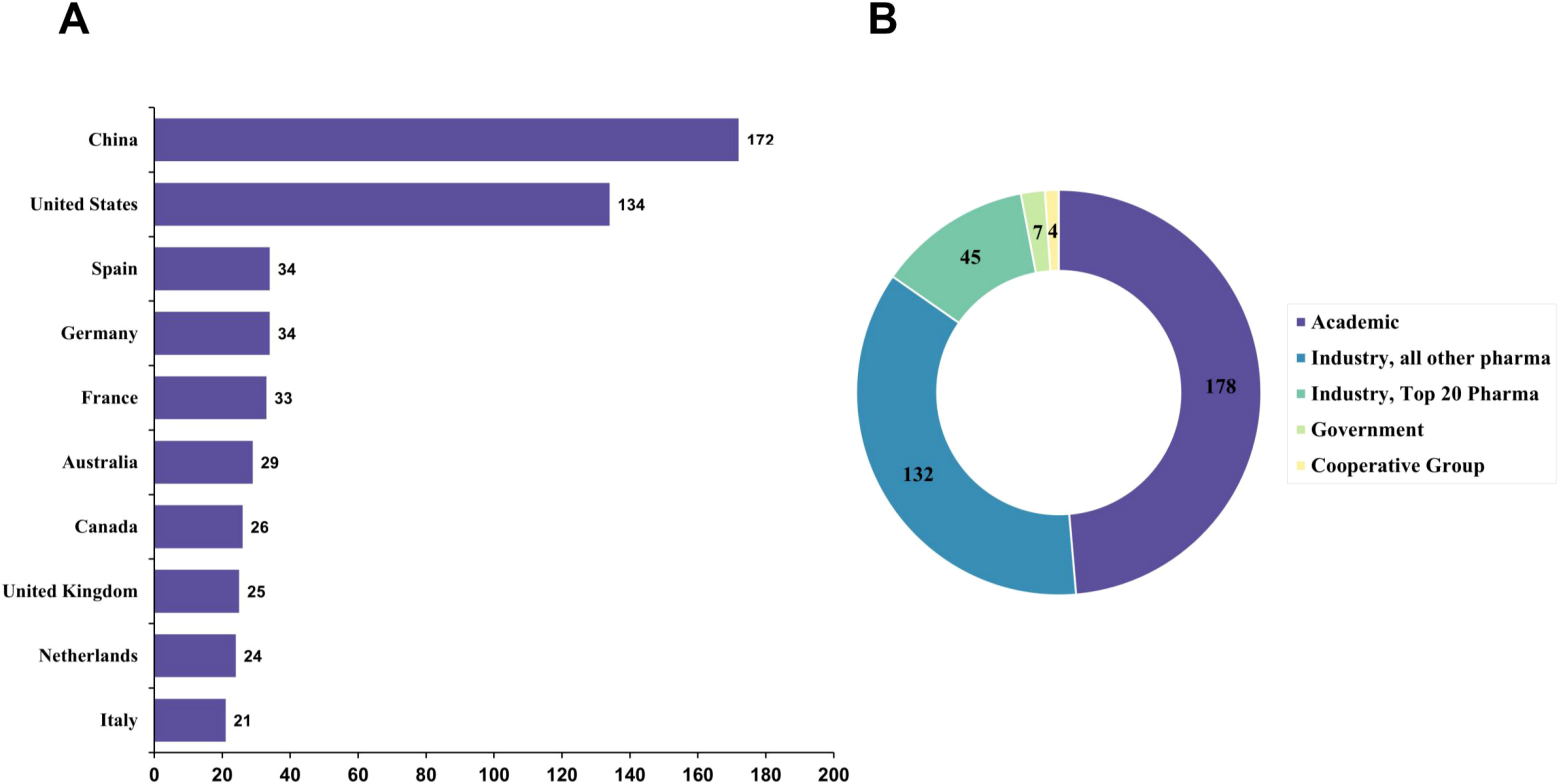
Top countries contributing to CAR-T clinical trials. **(A)** Bar chart showing the number of clinical trials conducted in the top contributing countries. China and the United States lead the global landscape, followed by several European countries and Australia. **(B)** Donut chart summarizing the distribution of sponsor types, highlighting the relative contributions of academic institutions and industry.

At the country level, China (172 trials) and the United States (134 trials) accounted for the largest share of registered studies, together representing the majority of worldwide trial activity. In contrast, European countries contributed more modest numbers of trials, including Spain (34 trials), Germany (34 trials), France (33 trials), and the United Kingdom (25 trials), with additional contributions from Australia (29 trials), Canada (26 trials), the Netherlands (24 trials), and Italy (21 trials) (**Figure 5A**). With respect to trial sponsorship, academic institutions represented the largest sponsor category, accounting for 178 trials, followed by industry-sponsored studies from non–top 20 pharmaceutical companies with 132 trials. Trials sponsored by top 20 pharmaceutical companies were less frequent (45 trials), while government-sponsored and cooperative group–led trials constituted only a small fraction of the overall landscape. This distribution suggests that CAR-T clinical development in NHL is predominantly led by academic centers, with substantial but secondary participation from industry sponsors (**Figure 5B**).

### 3.3 Molecular target landscape across NHL subtypes

**Figure 6:**
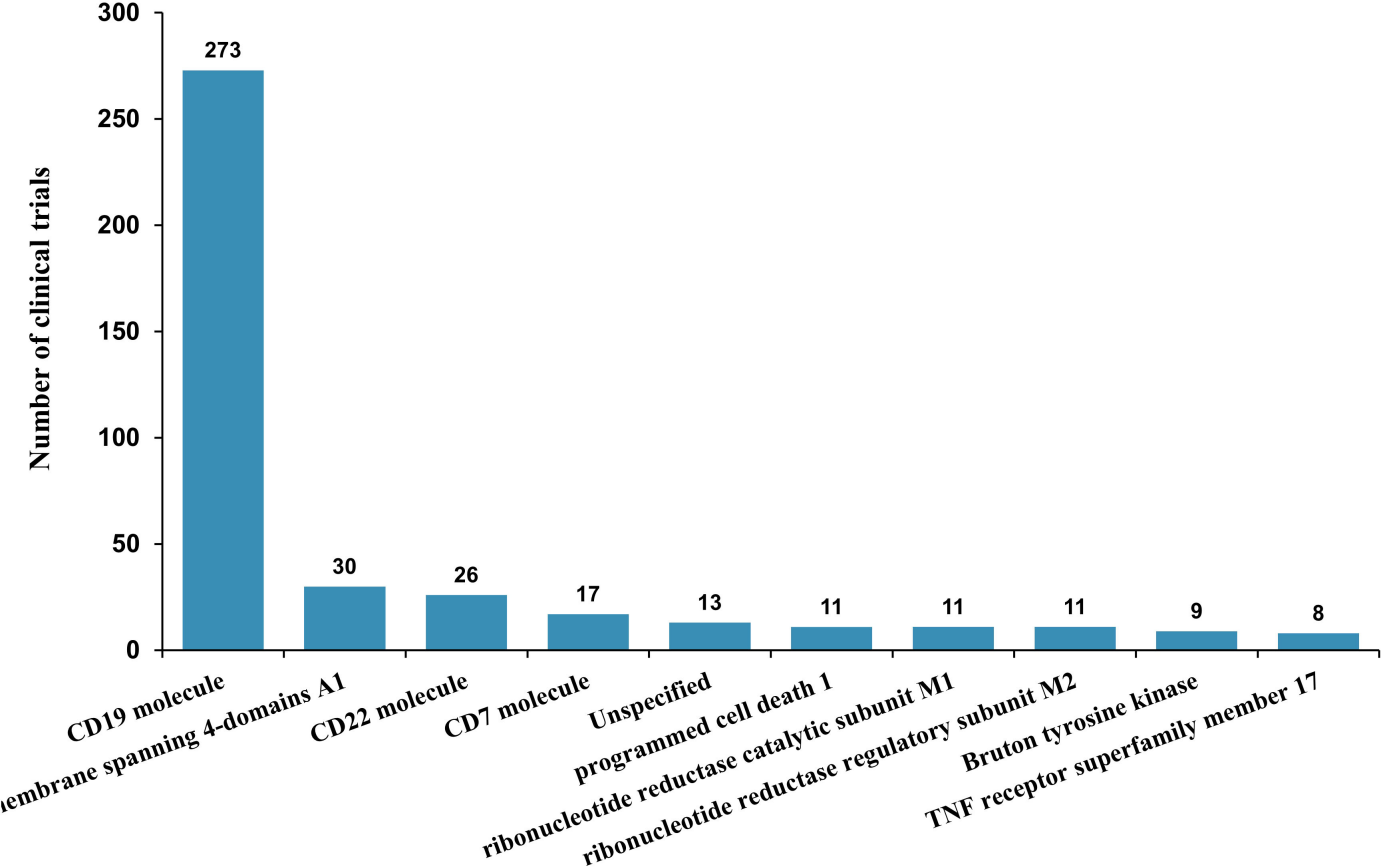
Frequency of CAR-T targets in clinical trials. The bar chart presents the number of clinical trials targeting specific antigens. CD19 is the most frequently targeted antigen, followed by CD22, CD7, and other emerging targets. Less frequently studied targets reflect ongoing exploration of novel therapeutic strategies.

The molecular target landscape of CAR-T clinical trials in non-Hodgkin lymphoma remains strongly CD19-centric, indicating that CD19-directed programs continue to anchor clinical development. In comparison, CD22, CD7, and MS4A1 (CD20) represent smaller but recurrent investigational themes, whereas the remaining targets are distributed across a broad long tail, reflecting exploratory efforts beyond canonical B-lineage antigens. Collectively, these findings suggest that, while the field is still dominated by a single established antigen, diversification toward alternative targets is increasingly visible within the registered trial portfolio (**Figure 6**).

**Figure 7:**
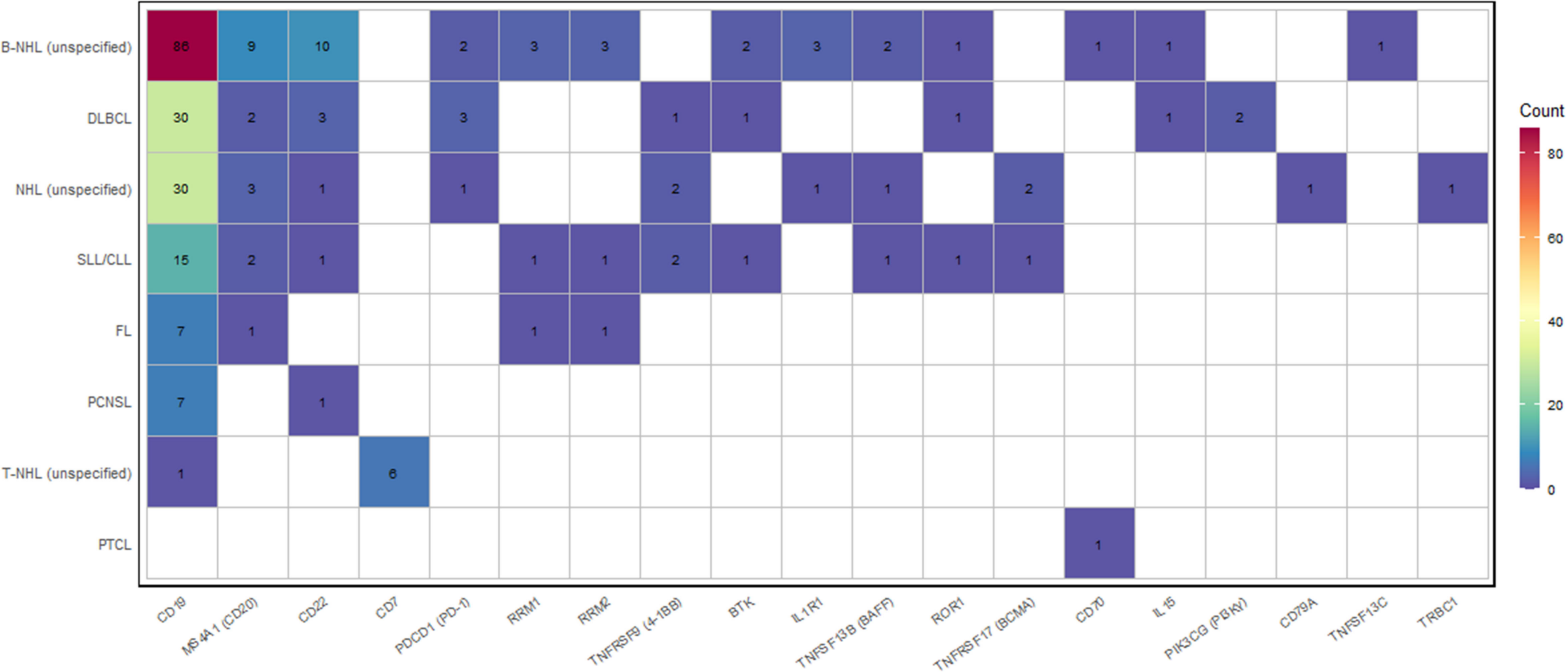
Distribution of CAR-T targets across NHL subtypes. The heatmap illustrates the frequency of different CAR-T targets across various NHL subtypes, including diffuse large B-cell lymphoma (DLBCL), follicular lymphoma (FL), small lymphocytic lymphoma/chronic lymphocytic leukemia (SLL/CLL), primary central nervous system lymphoma (PCNSL), and T-cell lymphomas. Color intensity corresponds to the number of trials for each antigen–subtype combination.

A subtype-resolved view further demonstrates lineage- and entity-specific target selection patterns. Across B-cell NHL categories, CAR-T development is primarily organized around B-lineage antigens (including CD19 and related B-cell targets), with additional exploratory testing of signaling or microenvironment-associated molecules in selected settings. In contrast, T-cell NHL categories are more closely linked to T-lineage targets, most prominently CD7, consistent with the biological context of T-cell malignancies. Trials registered under “unspecified” lymphoma categories contribute substantially to the breadth of targets observed, consistent with broader eligibility definitions and heterogeneous enrollment descriptions. Overall, the subtype–target matrix highlights both the dominance of B-lineage targeting in NHL and the emerging extension of CAR-T strategies into selected T-cell entities (**Figure 7**).

### 3.4 Clinical endpoints reported in CAR-T trials for NHL

**Figure 8:**
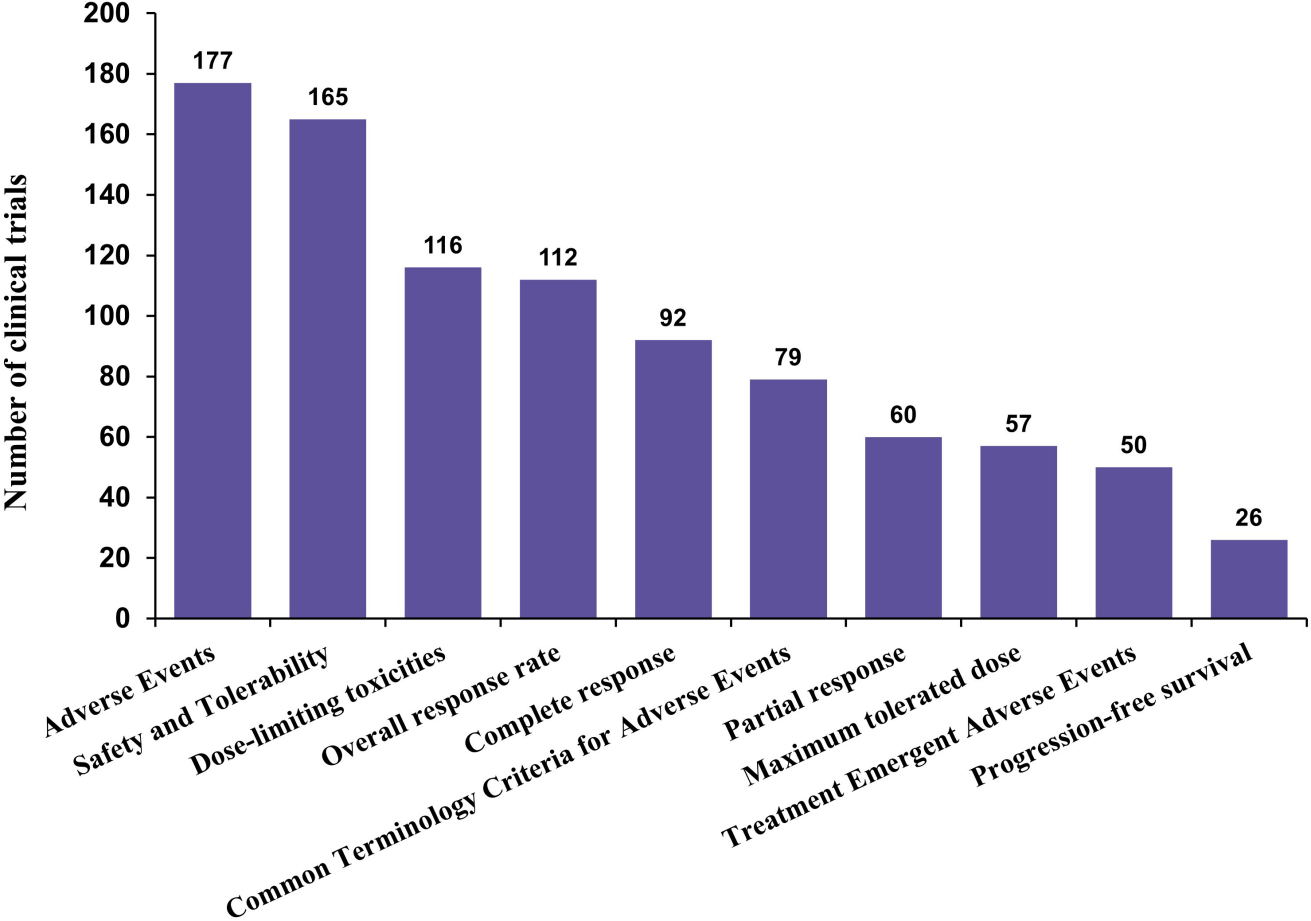
Distribution of reported clinical endpoints in CAR-T trials. The bar chart summarizes the frequency of reported clinical endpoints across included trials. Safety-related endpoints (e.g., adverse events, safety and tolerability, dose-limiting toxicities) are most commonly reported, whereas efficacy endpoints such as progression-free survival are less frequently captured, reflecting the early-phase nature of many studies.

Across CAR-T clinical trials in non-Hodgkin lymphoma, reported study endpoints were predominantly focused on safety-related outcomes. Adverse events, safety and tolerability, and dose-limiting toxicities were the most frequently assessed endpoints, underscoring the early-phase and risk-adaptive nature of CAR-T clinical development. Efficacy-related measures, including overall response rate (ORR) and complete response (CR), were also commonly reported, reflecting growing interest in preliminary antitumor activity alongside safety evaluation. In contrast, long-term clinical benefit endpoints were less frequently incorporated into trial designs. Measures such as progression-free survival (PFS) and treatment-emergent adverse events appeared in a smaller proportion of studies, while maximum tolerated dose and partial response were reported with intermediate frequency. Overall, the endpoint landscape indicates that CAR-T trials in NHL remain largely centered on safety characterization and short-term efficacy, with relatively limited emphasis on durable clinical outcomes (**Figure 8**).

## 4 Discussion

This Trialtrove-based landscape analysis provides a structured overview of contemporary CAR-T clinical development in NHL up to December 18, 2025. Several convergent signals emerge across the trial registry profile: rapid growth in trial activity over the past decade, a strong predominance of early-phase studies, geographic concentration in a limited number of countries, an academic-led sponsorship structure, CD19 as the dominant antigenic axis, and an endpoint portfolio largely weighted toward short-term safety and response assessments. Importantly, these findings collectively indicate that the current CAR-T clinical landscape in NHL is characterized by rapid expansion but remains predominantly early-stage, with innovation focused on optimizing efficacy, safety, and scalability rather than achieving broadly established long-term clinical benefit across all disease settings.

The clinical promise of CAR-T therapy in NHL is principally supported by mature datasets in relapsed or refractory large B-cell lymphoma (LBCL), where anti-CD19 products have produced high response rates and clinically meaningful durability in a subset of patients. Pivotal and long-term follow-up studies of axicabtagene ciloleucel (axi-cel), tisagenlecleucel (tisa-cel), and lisocabtagene maraleucel (liso-cel) consistently demonstrate deep remissions in heavily pretreated populations, establishing CAR-T as a transformative option for appropriate patients (16–18). At the same time, these studies also underscore a central limitation of first-generation CD19 CAR-T: durable benefit is not universal, and relapse remains an ongoing clinical problem, shaped by biological factors such as antigen escape and insufficient CAR-T persistence, as well as clinical factors including disease burden and patient fitness (19). Beyond third-line settings, CAR-T has moved earlier in the treatment paradigm for selected LBCL populations. Randomized phase 3 evidence has supported the use of axi-cel and liso-cel in the second-line setting for patients with early relapse or primary refractory disease, reinforcing the broader clinical momentum behind earlier deployment of cellular therapy in high-risk NHL (20, 21).

In parallel, the indication footprint of CAR-T in indolent and less common B-cell lymphomas is expanding (22, 23). For example, the U.S. FDA approved a new indication for lisocabtagene maraleucel as the first CAR-T therapy for marginal zone lymphoma in adults after at least two prior lines of therapy, highlighting ongoing extension beyond LBCL into additional NHL subtypes. While many registered trials in our dataset remain early phase and have not disclosed mature outcomes, the trajectory of regulatory decisions suggests that clinical translation is increasingly feasible when efficacy signals are strong and safety management is standardized.

The endpoint profile observed in the registry aligns with the practical realities of CAR-T development. Safety, adverse events, and tolerability remain the most frequently assessed outcomes, reflecting the need to optimize toxicity management and to standardize monitoring workflows as CAR-T expands beyond highly specialized centers (24). Although this study focuses on CAR-T therapies in NHL, broader class-wide regulatory developments across CAR-T platforms—including those targeting B-cell maturation antigen (BCMA) in related B-cell malignancies—provide important contextual insights. This focus is reinforced by evolving safety considerations at the class level. The U.S. Food and Drug Administration (FDA) has required boxed warning updates highlighting the risk of T-cell malignancies following BCMA- or CD19-directed CAR-T therapies and has recommended lifelong monitoring for secondary malignancies, emphasizing the importance of long-term pharmacovigilance and survivorship frameworks alongside efficacy evaluation. Europe has similarly mandated label updates and long-term monitoring recommendations, underscoring that late effects are now a central component of the benefit-risk assessment for CAR-T as a class. Notably, policy also reflects maturation of clinical practice. In June 2025, the FDA eliminated Risk Evaluation and Mitigation Strategies (REMS) requirements for currently approved autologous CD19- and BCMA-directed CAR-T therapies, indicating that risks are considered manageable through labeling and standard-of-care mitigation practices, which may reduce administrative burden and potentially streamline access. However, as registry data suggest that progression-free survival and other long-term endpoints remain less frequently prioritized than short-term response metrics, future trial designs may benefit from more consistent incorporation of durability endpoints, patient-reported outcomes, and standardized long-term follow-up, particularly as CAR-T is tested earlier in therapy lines where the clinical bar for durable benefit is higher (25).

The target distribution indicates that CD19 remains the central axis of innovation, but the field is actively diversifying. A major preclinical and early clinical thrust aims to mitigate antigen escape through dual-targeting or multi-targeting designs. Tandem or bicistronic CAR constructs targeting CD19 with CD20 or CD22 have been explored to broaden tumor coverage and reduce the likelihood of CD19-negative relapse, with early clinical experiences suggesting feasibility and encouraging antitumor activity (26–28). Alongside multi-antigen recognition, another prominent strategy is to augment CAR-T function within immunosuppressive tumor ecosystems using “armored” designs (29). IL-18 secreting CAR-T cells exemplify this approach, supported by preclinical evidence that IL-18 can enhance antitumor activity and by emerging early-phase clinical feasibility signals in lymphoma after prior CAR-T failure (30, 31).

Synthetic biology approaches are also expanding the conceptual toolbox for CAR-T in NHL. Logic-gated systems, including synthetic Notch (synNotch)-based circuits and related gating strategies, aim to improve tumor selectivity and safety by conditioning CAR expression or activation on multi-input antigen recognition (32). These designs may be particularly relevant when targeting antigens with broader normal tissue expression, or when moving CAR-T into settings where off-tumor risks must be minimized (33). Finally, access and manufacturing constraints have catalyzed interest in allogeneic, off-the-shelf cellular products, including allogeneic CAR-T and chimeric antigen receptor natural killer (CAR-NK) platforms, which may shorten time to treatment and improve scalability, although immune rejection and graft-related risks remain active challenges for the field (34–36). Taken together, these technological advances reflect a strategic shift from single-target, autologous approaches toward more versatile, multi-functional, and potentially scalable cellular therapies designed to address key limitations observed in first-generation CAR-T treatments.

Despite these advances, several recurrent challenges emerge from the current trial landscape. Antigen escape and limited CAR-T cell persistence remain major drivers of relapse, particularly in aggressive lymphoma subtypes. In parallel, treatment-related toxicities, including cytokine release syndrome and immune effector cell-associated neurotoxicity syndrome, continue to constrain broader clinical adoption. In addition, substantial heterogeneity in trial design, endpoint selection, and reporting standards limits cross-trial comparability and complicates evidence synthesis. Notably, the relative underrepresentation of long-term endpoints such as PFS and OS further highlights a gap between early efficacy signals and durable clinical benefit. These challenges underscore the need for both biological innovation and greater standardization in clinical trial design.

The marked geographic imbalance observed in this analysis, with a concentration of CAR-T clinical trials in high-income countries such as the United States and China, raises important concerns regarding global equity in access to advanced cellular therapies. Patients in low- and middle-income countries may face delayed access to CAR-T treatments due to limited clinical trial participation, insufficient infrastructure, and high treatment costs. Furthermore, the complex manufacturing process and logistical requirements associated with CAR-T therapy may further exacerbate these disparities. Without targeted efforts to expand clinical research networks and improve accessibility, there is a risk that the benefits of CAR-T therapy will remain unevenly distributed across global populations. Future strategies should prioritize more inclusive trial designs and scalable manufacturing approaches to address these challenges. The geographic clustering of trials and the predominance of academic sponsorship likely reflect a combination of innovation ecosystems, cellular therapy infrastructure, and regulatory pathways. Regions with established leukapheresis capacity, cell processing facilities, intensive monitoring units, and experienced multidisciplinary teams can more readily initiate and sustain CAR-T trials. At the same time, regulatory and policy frameworks have direct effects on trial velocity, postapproval monitoring expectations, and access. The combination of strengthened long-term safety labeling for secondary malignancies and the removal of REMS requirements illustrates a dual policy signal: safety oversight is tightening in terms of long-term surveillance, while operational barriers may be easing to facilitate broader delivery of established products. These shifts are likely to influence endpoint selection and follow-up strategies in future trials, and they emphasize the importance of harmonizing trial design with real-world implementation needs, including survivorship monitoring and system-level readiness.

Several limitations should be considered when interpreting this landscape analysis. First, Trialtrove integrates information from multiple registries and sources, but registry records may be incomplete, inconsistently updated, or heterogeneous in terminology, particularly for targets, subtype definitions, and endpoints. Second, a key limitation of this study is the lack of patient-level data, such as age, disease stage, and prior treatment history, which are not consistently reported in clinical trial registries. As a result, our analysis is restricted to trial-level characteristics and does not capture intra-trial heterogeneity. Future studies integrating patient-level datasets or detailed trial publications are warranted to provide more granular clinical insights. Third, some trials disclose results in publications or conferences without fully updating registry fields, whereas other trials may be registered but never proceed to enrollment, introducing uncertainty when using registration as a proxy for active development. Fourth, our rule-based harmonization for subtype and target classification, while designed to be conservative and to avoid over-inference, may still misclassify ambiguous entries or underrepresent complex multi-target designs. Finally, because this study emphasizes descriptive patterns rather than comparative effectiveness, conclusions should be interpreted as developmental signals rather than evidence of superiority between products or strategies.

Taken together, the registry landscape suggests that CAR-T therapy in NHL is transitioning from an era dominated by first-generation CD19 products in late-line LBCL toward a broader developmental portfolio, including earlier-line testing, expanded subtype coverage, and next-generation engineering aimed at durability, safety, and scalability. Future directions should focus on both technological innovation and clinical standardization. From a biological perspective, multi-antigen targeting strategies, armored CAR-T constructs, and gene-edited allogeneic platforms represent promising approaches to improve efficacy, durability, and scalability. From a clinical standpoint, greater emphasis on standardized endpoint definitions, incorporation of long-term follow-up measures, and integration of patient-reported outcomes will be essential to better capture real-world therapeutic value. Furthermore, combining CAR-T therapy with targeted agents or immune modulators may provide synergistic benefits and help overcome resistance mechanisms. Importantly, improved integration of registry data with patient-level datasets and published trial outcomes will be critical for generating more granular and clinically actionable insights.

## 5 Conclusion

This clinical trial landscape analysis highlights the rapid expansion and evolving complexity of CAR-T cell therapy development in NHL. Current clinical efforts remain largely concentrated on early-phase studies, with a strong emphasis on safety assessment and short-term efficacy, and continued dominance of CD19-directed strategies. Meanwhile, emerging diversification in target selection, CAR design, and preclinical innovation reflects growing recognition of key challenges such as antigen escape, limited persistence, and treatment-related toxicities. Future progress will depend on the integration of next-generation CAR engineering, rational trial design incorporating durable clinical endpoints, and alignment with regulatory and health policy frameworks to facilitate broader and more sustainable clinical translation of CAR-T therapies in NHL.

## Data Availability

The raw data supporting the conclusions of this article will be made available by the authors, without undue reservation.
