# Correction: CAR-T cell therapy in non-Hodgkin lymphoma: a clinical trial landscape review

**DOI:** 10.3389/fimmu.2026.1879167

**Published:** 2026-05-25

**Authors:** Jinyu Wu, Yun Liao, Wen Wang, Xin Zhang

**Affiliations:** 1 Department of Gastroenterology, The First People’s Hospital of Shuangliu District (West China Airport Hospital of Sichuan University), Chengdu, Sichuan, China; 2 Stem Cell Immunity and Regeneration Key Laboratory of Luzhou, Sichuan, Luzhou, China; 3 Department of Laboratory Medicine, The Second Affiliated Hospital of Chengdu Medical College, Nuclear Industry 416 Hospital, Chengdu, Sichuan, China

**Keywords:** chimeric antigen receptor T cell, clinical trial landscape, immunotherapy, molecular targets, non-Hodgkin lymphoma

There was a mistake in **Figure 3** as published. Due to an inadvertent upload error during the submission/production process, **Figure 3** was incorrectly duplicated from **Figure 5B**.

**Figure 3:**
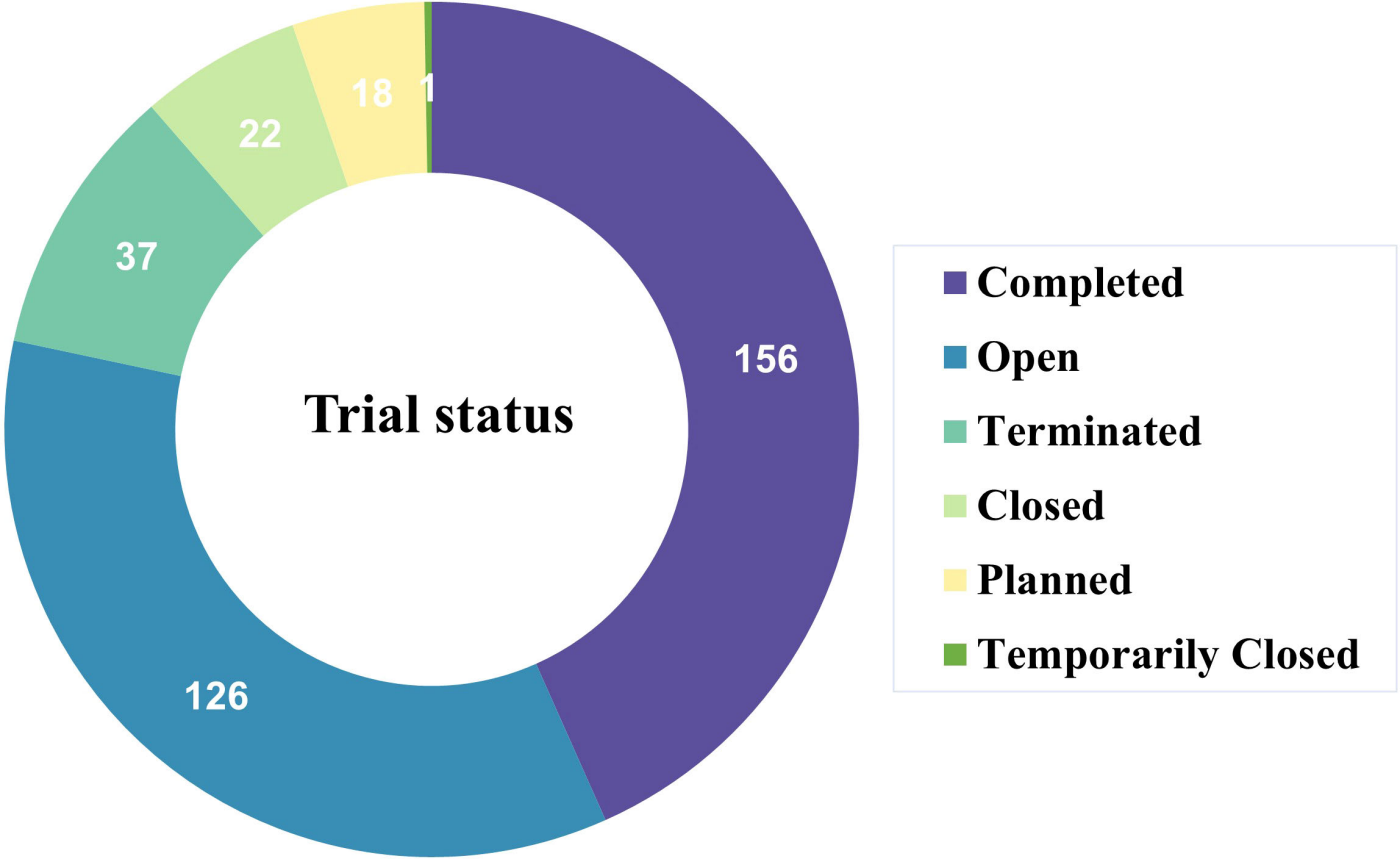
Distribution of trial status among registered CAR-T clinical trials for non-Hodgkin lymphoma. The donut chart summarizes the operational status of the included trials identified from the Trialtrove database. Most studies were classified as completed or actively open, whereas smaller proportions were terminated, closed, planned, or temporarily closed. These findings reflect the ongoing expansion and dynamic evolution of CAR-T clinical development in NHL.

The corrected **Figure 3** and caption appear below.

**Figure 3**. Distribution of trial status among registered CAR-T clinical trials for non-Hodgkin lymphoma. The donut chart summarizes the operational status of the included trials identified from the Trialtrove database. Most studies were classified as completed or actively open, whereas smaller proportions were terminated, closed, planned, or temporarily closed. These findings reflect the ongoing expansion and dynamic evolution of CAR-T clinical development in NHL.

The original version of this article has been updated.

